# Interspecies Interaction between *Pseudomonas aeruginosa* and Other Microorganisms

**DOI:** 10.1264/jsme2.ME12167

**Published:** 2013-01-30

**Authors:** Yosuke Tashiro, Yutaka Yawata, Masanori Toyofuku, Hiroo Uchiyama, Nobuhiko Nomura

**Affiliations:** 1 Division of Environmental Engineering, Faculty of Engineering, Hokkaido University, North-13, West-8, Kita-ku, Sapporo, Hokkaido, 060–8628, Japan; 2 Department of Civil and Environmental Engineering, Massachusetts Institute of Technology, Cambridge, MA, USA; 3 Graduate School of Life and Environmental Sciences, University of Tsukuba, 1–1–1 Tennodai, Tsukuba, Ibaraki 305–8572, Japan

**Keywords:** *Pseudomonas aeruginosa*, quorum sensing, cross-talk, membrane vesicles, micro device

## Abstract

Microbes interact with each other in multicellular communities and this interaction enables certain microorganisms to survive in various environments. *Pseudomonas aeruginosa* is a highly adaptable bacterium that ubiquitously inhabits diverse environments including soil, marine habitats, plants and animals. Behind this adaptivity, *P. aeruginosa* has abilities not only to outcompete others but also to communicate with each other to develop a multispecies community. In this review, we focus on how *P. aeruginosa* interacts with other microorganisms. *P. aeruginosa* secretes antimicrobial chemicals to compete and signal molecules to cooperate with other organisms. In other cases, it directly conveys antimicrobial enzymes to other bacteria using the Type VI secretion system (T6SS) or membrane vesicles (MVs). Quorum sensing is a central regulatory system used to exert their ability including antimicrobial effects and cooperation with other microbes. At least three quorum sensing systems are found in *P. aeruginosa*, Las, Rhl and *Pseudomonas* quinolone signal (PQS) systems. These quorum-sensing systems control the synthesis of extracellular antimicrobial chemicals as well as interaction with other organisms via T6SS or MVs. In addition, we explain the potential of microbial interaction analysis using several micro devices, which would bring fresh sensitivity to the study of interspecies interaction between *P. aeruginosa* and other organisms.

## Introduction

For a long period, microbiology developed based on pure culture studies and these oversimplified analyses contributed to the understanding of the complex functions of such small organisms; however, a variety of microbial species coexist in the natural environment and interact with each other, building an advanced network society (40, 56, 89). Given that, for example, as many as several million species of bacteria exist in 1 g of pristine soil (37), the microbial society in the natural environment must be much more complicated than what is observed under laboratory conditions. Microbial interactions are roughly classified into ‘competition’ and ‘cooperation’. Over the years, our knowledge about microbial competition has undoubtedly developed by the study of antimicrobials. Synthesis and secretion of antimicrobial chemicals are sophisticated strategies for microorganisms to achieve their ecological niches in multi-species coexisting environments. Understanding cooperative and synergistic bacterial interactions has recently undergone marked growth by studies of quorum sensing, cross-talk and electron transfer in microbial communities such as biofilms. Bacterial interaction through signal molecules or minerals allows microbial cells to coordinate group activity, which ensures stronger and more efficient ways to survive in a hostile environment than individual activity.

*Pseudomonas aeruginosa* is a highly adaptable bacterium that can colonize various environmental niches, including soil, marine habitats, plants and animals (58, 60, 115). This bacterium is also known as an opportunistic human pathogen, causing infections of the eyes, ear, skin, urethra and respiratory tract in cystic fibrosis (CF) and burn patients, as well as other immunocompromised individuals. In addition, *P. aeruginosa* causes diseases in plants and animals. Thus, this bacterium is known to be highly adaptable and to have diverse phenotypic characteristics, which enable it to inhabit various environmental conditions.

**Fig. 1:**
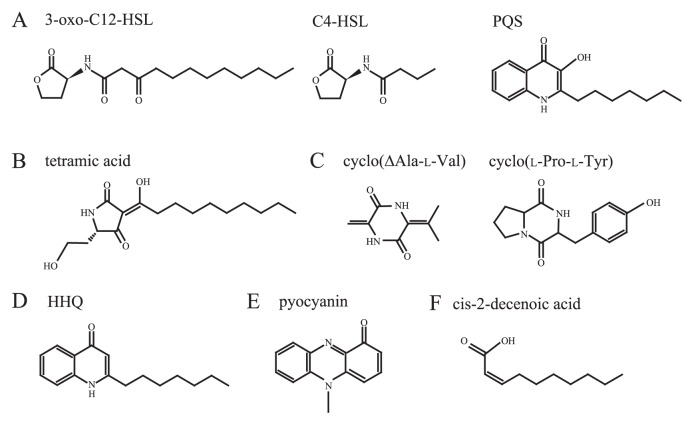
Chemical structures of signals secreted by *P. aeruginosa*. (A) Three autoinducers. (B) A tetramic acid, 3-(1-hydroxydecylidene)-5-(2-hydroxyethyl)pyrrolidine-2,4-dione, which is a degraded compound of 3-oxo-C12-HSL. (C) Two cyclic dipeptides (2,5-diketopiperazines: DKPs), cyclo(ΔAla-l-Val) and cyclo(l-Pro-l-Tyr). (D) HHQ, a precursor of PQS. (E) Pyocyanin, one of phenazine. (F) cis-2-decenoic acid, a fatty acid signal.

**Fig. 2:**
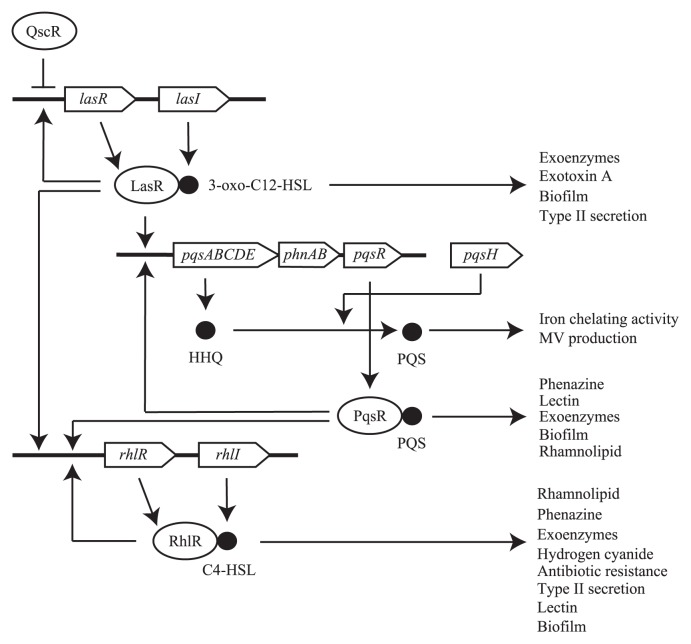
An overview of QS hierarchy model in *P. aeruginosa. P. aeruginosa* has three quorum-sensing systems, Las, Rhl and PQS. In the Las system, a receptor, LasR, is activated by its cognate AHL, 3-oxo-C12-HSL, synthesized by LasI, and the complex activates Las, PQS and Rhl systems. PQS binds its receptor, PqsR, and the complex regulates PQS and Rhl systems. The Rhl system consists of RhlR and its cognate signal, C4-HSL, synthesized by RhlI and the complex autoregulates the Rhl system. These quorum-sensing systems control the production of a range of virulence factors and various behaviors.

One of the reasons why *P. aeruginosa* inhabits various environments ubiquitously seems to be because it has specific functions to interact with other bacteria. A quintessential system, which contributes to its interspecies interaction, is quorum sensing. In this system, bacteria produce and release chemical signal molecules called autoinducers (AIs), that increase in concentration as a function of cell density, and they regulate gene expression in response to fluctuations in cell-population density (80, 92). *P. aeruginosa* has at least three type quorum-sensing systems, Las, Rhl and Pqs (*Pseudomonas* quinolone signal). *N*-(3-oxo-dodecanoyl) l-homoserine lactone (3-oxo-C12-HSL), *N*-butanoyl l-homoserinelactone (C4-HSL) and 2-heptyl-3-hydroxy-4-quinolone (PQS) are used as AIs in these systems, respectively (Figs. 1A and 2). These quorum-sensing systems regulate the synthesis of several virulence factors, including pyocyanin and rhamnolipids in *P. aeruginosa*. These virulence factors were traditionally thought to be antimicrobials but a recent study shed light on other functions of these molecules to control bacterial group behavior. In addition, *P. aeruginosa* effectively transferred specific proteins to other bacteria via the type VI secretion system (T6SS) or membrane vesicles (MVs). These systems may contribute to the lysis of other microbes to achieve niches in poly-microbial communities. This review summarizes knowledge on microbial interspecies interactions between *P. aeruginosa* and other organisms. Moreover, we also suggest that micro-fabrication technology would be powerful tools to develop the understanding of such microbial interactions.

## Interspecies interaction via N-acylhomoserine lactones

### *N*-acylhomoserine lactones can be interspecies signals

*P. aeruginosa* uses two *N*-acylhomoserine lactones (AHLs), 3-oxo-C12-HSL and C4-HSL (Fig. 1A), that bind LuxR-type intracellular receptor proteins, LasR and RhlR, respectively. AHLs are secreted and their concentration rises as the population grows, enabling the bacterium to monitor cellular density. Several virulence factors are regulated by this system (Fig. 2). Given that bacteria other than *P. aeruginosa* produce these signal molecules, these AIs can potentially function as cross-talk signals between *P. aeruginosa* and other bacteria. For example, *Yersinia enterocolitica* produces 3-oxo-C12 HSL as one of the by-products via LuxI homologue YenI, although this bacterium mainly uses 3-oxo-C6-HSL and C6-HSL (4). C4-HSL is used as a quorum-sensing signal in *Aeromonas hydrophila*, *Aeromonas salmonicida*, *Serratia liquefaciens*, *Serratia* sp. ATCC39006 and *Vibrio vulnificus* (32, 116, 125, 133).

It is known that some LuxR proteins of other bacteria recognize a variety of AHLs (81, 110, 151). In the case of *P. aeruginosa*, the responses, particularly of LasR, to AHLs are relatively specific (109). A third LuxR protein, QscR, which has a negative effect on some genes of other quorum-sensing systems (20, 63), broadly binds to C10 HSL, 3-oxo-C10-HSL and C12-HSL as well as 3-oxo-C12-HSL, although the binding affinities are much lower than that of LasR to 3-oxo-C12-HSL (65). These results raise the possibility that *P. aeruginosa* communicate with other bacteria.

**Fig. 3:**
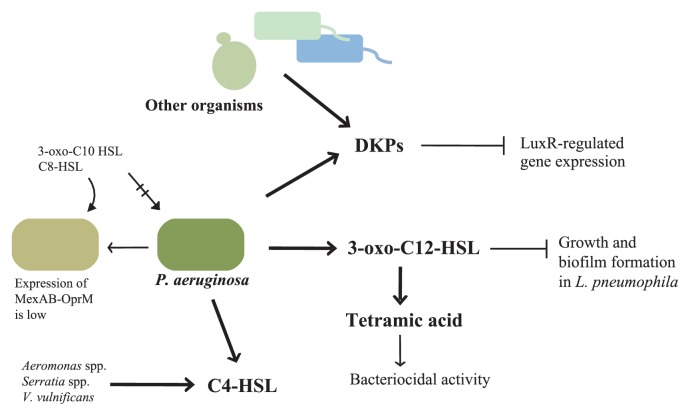
Interaction between *P. aeruginosa* and other microorganisms mediated by AHLs and DKPs. *P. aeruginosa* uses 3-oxo-C12-HSL and C4-HSL as quorum-sensing signals. 3-oxo-C12-HSL is degraded to tetramic acid, which has bacteriocidal activity. Many organisms as well as *P. aeruginosa* secrete DKPs, which repress LuxR-type quorum sensing including Las and Rhl. When an efflux pump MexAB-OprM does not function, *P. aeruginosa* can alter phenotypes by other AHLs.

On the other hand, some data suggest that *P. aeruginosa* has other mechanisms than the LuxR specificity to select AIs and may tend to use mainly their own AIs and eliminate other signals. For example, *Burkholderia cepacia*, which synthesizes C8-HSL as its AI, can perceive the AHLs produced by *P. aeruginosa* in mixed *P. aeruginosa/B. cepacia* biofilm cultures *in vitro* and *in vivo* in a mouse chronic lung infection model, but not vice versa (102). Hence, it is considered that *P. aeruginosa* has a system to detect a specific length of AHLs. Crystal structure analysis of the N-terminal half of LasR suggested that the acyl side-chain length of AHLs is not the main factor that determines the specificity of receptor protein binding (8); therefore, it has been a mystery how *P. aeruginosa* recognizes the difference in the length of acyl-chains. A recent report shed light on the underlying reasons. Minagawa *et al.* (82) showed that the RND-type efflux pump system MexAB-OprM controls the accessibility of non-cognate AHLs to LasR but not the binding of LasR, and therefore *P. aeruginosa* has the ability to select its signal language. Indeed, MexAB-OprM deletion mutant showed a strong quorum-sensing response to 3-oxo-C10-HSL and cross-talked with *Vibrio anguillarum* (82). In addition, MexAB-OprM deletion mutant responded to C8-HSL and cross-talked with *B. cepacia*, but this reaction was not via known LuxR-type receptors (145). In this way, MexAB-OprM mutant has potential to cross-talk with other bacteria via AHL other than 3-oxo-C12-HSL and C4-HSL (Fig. 3). Interestingly, it has been shown that the expression of MexAB-OprM is decreased in biofilm (25). Although it has been reported that *P. aeruginosa* could not detect C8-HSL from *B. cepacia* in mixed biofilm (102), it remains a possibility that *P. aeruginosa* recognize more than two AHLs under different conditions in mixed biofilm consisting of multibacterial species.

### Antimicrobial effects of AHLs and their degradative products

Recently, Kimura *et al.* (61) have reported that the 3-oxo-C12-HSL molecule itself also has competitive effects against other bacteria. 3-oxo-C12-HSL specifically suppresses growth and biofilm formation in *Legionella pneumophila*, although it does not show bacteriocidal or bacteriostatic actions against other Gram-negative bacteria that the authors tested. However, it was found that a tetramic acid, 3-(1-hydroxydecylidene)-5-(2-hydroxyethyl)pyrrolidine-2,4-dione (Fig. 1B), which is a degradation product of 3-oxo-C12-HSL, has innate bactericidal activities against all tested Gram-positive bacteria, such as *Bacillus cereus* and *Staphylococcus aureus*, by dissipating membrane potential and transmembrane proton motive force (57, 70). Tetramic acid is spontaneously produced from 3-oxo-C12-AHL and found in *P. aeruginosa* culture supernatants. Hosono *et al.* (48) showed that a high concentration (100 μM) of tetramic acid also has antimicrobial activities against Gram-negative bacteria, including *Escherichia coli* and *Klebsiella pneumonia*, as well as *P. aeruginosa* itself. The authors suggested that these high concentrations may be biologically meaningful in certain situations, because high concentrations of 3-oxo-C12-HSL (>600 μM) have been detected in *P. aeruginosa* biofilms. In this way, the degradation product of 3-oxo-C12-HSL induces self-killing and precluding other bacteria (Fig. 3).

### Cyclic dipeptides are inhibitors of AHL-based quorum sensing

The response of LuxR is not limited to AHLs. Holden *et al.* (45) have reported that *P. aeruginosa*, as well as *Proteus mirabilis*, *Citrobacter freundii* and *Enterobacter agglomerans*, secretes two cyclic dipeptides (2,5-diketopiperazines: DKPs), cyclo(ΔAla-l-Val) and/or cyclo (l-Pro-l-Tyr) (Fig. 1C) and these DKPs antagonize 3-oxo-C6-HSL-mediated induction of bioluminescence and activate the C4-HSL-dependent swarming motility of *Serratia liquefaciens*. Similar, but not structurally identical, DKPs are secreted by other Gram-negative bacteria, Gram-positive bacteria, archaea, fungi and higher organisms, and these signal molecules affect LuxR-mediated phenotypes (26, 45, 126, 135). More recently, Campbell *et al.* (12) synthesized DKPs, which inhibit quorum sensing-regulated bioluminescence; however, these chemically synthesized DKPs did not interact with LuxR directly. In this way, a conclusive answer has not been found regarding how DKPs interact with AHL-based quorum sensing; however, these studies indicate that DKPs have a role in interspecies signaling to affect AHL-based quorum sensing (Fig. 3).

## Quinolone signaling in *Pseudomonas aeruginosa*

### 2-alkyl-4-qionolone signaling in bacteria

**Fig. 4:**
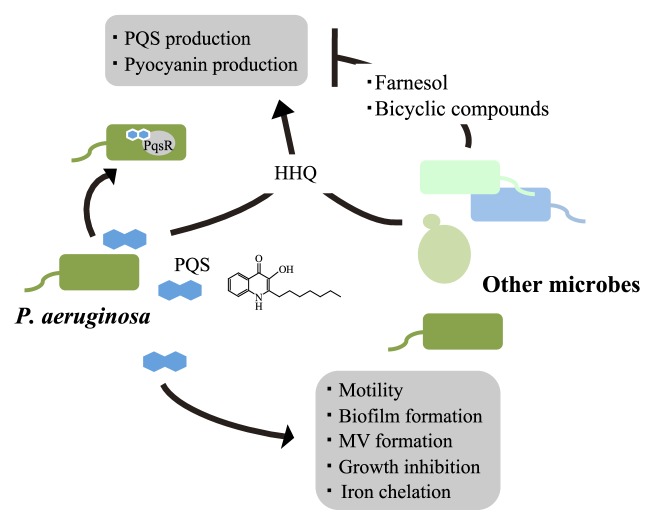
Interaction between *P. aeruginosa* and other microorganisms mediated by PQS. In *P. aeruginosa*, PQS works as a cell-cell communication signal that regulates the expression of genes via its cognate receptor PqsR. In addition, PQS affects several biological processes, such as motility, biofilm formation, MV formation in *P. aeruginosa* and other microorganisms that do not require PqsR. Other micro-organisms may interfere with PQS production via HHQ, farnesol and bicyclic compounds such as indole.

PQS is one of the primary quorum-sensing signals as well as the two AHLs in *P. aeruginosa* (93). This signal is recognized by its cognate receptor PqsR and regulates the production of PQS as well as virulence factors such as elastase, rhaminolipids and pyocyanin (36) (Fig. 2). The direct precursor of PQS, 2-heptyl-4-quinolone (HHQ) (Fig. 1D), along with other chemically related 2-alkyl-4-qionolones (AQs) are produced from the product of the *pqsABCDE* operon and such AQs are detected in the supernatant together with PQS (27). HHQ is further converted to PQS by PqsH, which is a monooxygenase whose gene is located at a distinct locus from the *pqsABCDE* operon (36). Besides PQS, HHQ also functions as a signaling molecule by binding to PqsR and induces *pqsA* transcription; however, the binding affinity of HHQ to PqsR is much lower than PQS, suggesting that PQS is the major signal in *P. aeruginosa* (141). Although PQS is considered as the major signal under aerobic conditions, HHQ may play important roles under anaerobic conditions. Since PqsH is a monooxygenase that requires oxygen for PQS synthesis, PQS is not produced under anaerobic conditions (129). As a consequence, HHQ accumulates under anaerobic conditions (107), although its function as a signaling molecule under these conditions has not been examined yet in detail. Further studies have demonstrated that AQs other than PQS play central roles in cell-to-cell communication in certain bacteria. To date, PQS is only produced by *P. aeruginosa* while HHQ is also produced in *Pseudomonas putida* (30, 134). A further study revealed that *Burkholderia* species produce methylated AQs (134). While the receptor for AQs in these organisms has not been characterized, it was shown in *Burkholderia pseudomallei* that the defect in AQ production alters its colony morphology, suggesting that AQ(s) functions as a signaling molecule in this bacteria (30). Once HHQ accumulates in the environment, *P. aeruginosa* can take up these HHQ and alter its gene expression (27). Taken together, AQs such as HHQ can be considered as an intra and inter-species communication signal, while PQS has evolved as a *P. aeruginosa*-specific signal by acquiring PqsH (Fig. 4).

### Multifunctional PQS

Besides its function as a signaling molecule, PQS is a multifunctional molecule that chelates iron and also plays a role in redox homeostasis (Fig. 4). Rapid loss of free iron was observed when PQS was added to the medium where PQS chelates Fe (III) in a 3:1 complex (9, 31). In redox homeostasis, PQS has been demonstrated to work both as an anti-oxidant and pro-oxidant. While PQS is primarily an anti-oxidant, the presence of iron induces a pro-oxidant effect (41). These functions based on the chemical characteristics of PQS alter the gene expression of *P. aeruginosa* and other micro-organisms in a PqsR-dependent manner. In this manner, PQS acts as a stimulant rather than a signal.

PQS has also been demonstrated to induce MV production and PQS itself is packaged and potentially trafficked by these MVs (73). MVs are mainly composed of an outer membrane and are secreted from the bacterial cell surface as a delivery vehicle. A previous report has demonstrated that approximately 90% of PQS is associated with MVs (73). In addition to PQS, related AQs including HHQ and 4-hydroxy-2-heptylquinoline-*N*-oxide (HQNO) are also detected in MVs produced by *P. aeruginosa* (73). Further research demonstrated that this PQS localization could be altered by inactivation of a periplasmic protein MucD protease (122), implying that an unknown mechanism may be involved in the PQS sorting to MVs. The induction of MV production by PQS is suggested whereby PQS interact with the membrane of the bacteria. PQS insertion into the membrane leads to asymmetric growth of the outer leaflet of the lipid bilayer membrane that results in the budding of the membrane (74, 108). In line with this evidence, when PQS is added to other bacteria, it induces MV formation in other Gram-negative bacteria such as *E. coli* and even in Gram-positive bacterium *Bacillus subtilis* (118). Taking into account the biological functions of MVs, the presence of PQS could enhance interactions among bacteria as well as the interactions of bacteria with higher organisms.

### Influence of AQs on bacteria

As discussed above, AQs including PQS are multifunctional molecules that potentially could affect other bacteria (Fig. 4). A *pqsA* mutant of *P. aeruginosa* was shown to lose its effect on lyses of *S. aureus*, suggesting that AQs or genes regulated by AQs are involved in this *S. aureus* lysis (72). On the other hand, the presence of *P. aeruginosa* protects *S. aureus* from aminoglycosides, which is mediated by HQNO (44). HQNO is generally known to inhibit respiration, which is involved in the uptake of aminoglycosides; therefore, it is suggested that HQNO suppress respiration, which result in aminoglycoside resistance of *S. aureus* (44). In this report, the authors further showed that HQNO selects for *S. aureus* small-colony variants that are observed in diverse infections, implying that these interactions among bacteria impact the community structure of bacteria and further the health of the host.

Besides HQNO, PQS also inhibits respiration. *P. aeruginosa* utilize N-oxides instead of oxygen as a terminal electron acceptor, a process known as denitrification (130). Along with nitrification, which oxidizes ammonia to NO_3_^−^(85, 111), these processes are important in nitrogen cycling (51, 77, 90). Our studies along with others have revealed that quorum sensing regulates denitrification (128, 150). AHLs and PQS regulate denitrification in a different manner. AHLs regulate the transcription of denitrifying genes via their LuxR family transcriptional regulators, while the PQS effects on denitrification are partially PqsR dependent and mainly act post-transcriptionally in a PqsR-independent manner. Further evidence indicated that PQS acts directly on denitrifying enzymes, particularly on NO_3_^−^ respiratory enzymes and nitric oxide reductase (NOR) (129). This was the first study to demonstrate that a signaling molecule affects enzyme activity in a direct manner. It is assumed that PQS iron-chelating activity is a key in the inhibition of denitrification by PQS since excess amounts of iron inhibit the effect of PQS on denitrification (129).

Further study on PQS demonstrated that similar effects on growth and respiration are also observed against phylo-genetically distinct groups of bacteria (127). PQS affected the growth and oxygen consumption rate of *E. coli* and *P. putida*, suggesting that PQS may inhibit the respiratory chain, similar to what was observed in the PQS effect on denitrification. When PQS was added to a bacterial community isolated from soil, PQS affected the growth of many but not all Gram-negative and Gram-positive bacteria (127). The effect of PQS on bacterial growth was not like that of antibiotics that inhibit growth or induce cell lysis, but rather slowed the growth rate. The influence of PQS was not observed in the presence of excess iron, indicating that the iron concentration is the key in such interactions. The precise mechanism of how PQS inhibits growth in some bacteria and why other bacteria tolerate growth inhibition by PQS are still unclear. More recently, it was revealed that PQS and HHQ modulate various phenotypes, including motility and biofilm formation in Gram-negative and Gram-positive bacteria as well as yeasts (34, 100). These wide impacts of PQS and other AQs on micro-organisms may be effective in shaping the community structure and altering the function of the community.

### Interference in PQS signaling by other micro-organisms

As PQS is multifunctional and influences various phenotypes in a broad range of micro-organisms, some micro-organisms avoid this effect by inhibiting PQS production (Fig. 4). For example, *Candida albicans* is a fungal pathogen that is commonly found with *P. aeruginosa* in infections. When *C. albicans* and *P. aeruginosa* are co-cultured, reduced pyocyanin production, a phenotype regulated by PQS in *P. aeruginosa*, is observed, suggesting an interaction among these micro-organisms (22). In the same study, it was further demonstrated that farnesol affects PQS signaling in *P. aeruginosa*. In *C. albicans*, farnesol is used as a cell-to-cell communication signal that regulates hyphal formation. Farnesol is suggested to affect PQS signaling via a mechanism that involves direct binding with PqsR. While farnesol stimulates PqsR binding to the target promoter of *pqsA*, it does not induce transcription of *pqsA*, leading to the inhibition of PQS production. Interactions between *P. aeruginosa* and *Candida* species have been broadly observed, suggesting complex bacterial-fungal interactions (5, 22, 38). Another signal, indole, which is used as a signaling molecule in *E. coli* and many other bacteria (43), also represses PQS signaling, but the mechanism is still unknown (64). Recently it was demonstrated that when *P. aeruginosa* and *E. coli* were co-cultured, *E. coli* growth was promoted in an indole-dependent manner (19). Although it was not studied further, this may have been due to PQS production inhibition by indole. Furthermore, structurally related bicyclic compounds were also shown to inhibit PQS signaling and MV production (123). These bicyclic compounds, such as hydroxyindole and isatin, are produced by a number of bacteria, including *Pseudomonas* and *Burkoholderia* species (33, 103, 149). Given these results, PQS signaling can be modulated in the presence of other micro-organisms, which will be important in understanding how bacteria communicate in the real world where they survive in a complex community (117, 131). To date, little is known about how PQS is degraded and future work may isolate PQS degraders, as in the case of AHLs (86, 99, 138)

## Phenazines are antimicrobials, interspecies signals and electron transfer mediators

Phenazine compounds are colorful secondary metabolites synthesized by various fluorescent *Pseudomonas* species, such as *P. aeruginosa*, *Pseudomonas fluorescens* and *Pseudomonas aureofaciens*. Their biosyntheses have also been reported in bacteria belonging to other families and classes, and even archaea (94). Phenazine compounds are known as redox-active and pigmented antibiotics. *P. aeruginosa* produces a variety of phenazine compounds, including pyocyanin (Fig. 1E), phenazine-1-carboxylic acid (PCA), 1-hydrox-yphenazine (1-OH-PHZ), and phenazine-1-carboxamide (PCN), which synthesize under quorum-sensing regulations. Phenazines have toxicity against bacteria and fungi and it is thought that this is due to the generation of reactive oxygen species (94). In rhizospheres, many kinds of *Pseudomonas* species, such as *P. aureofaciens*, *P. fluorescens* and *Pseudomonas chlororaphilus*, exist surrounding the roots of plants and form microbial communities (113). In these complexed rhizospheres, phenazine is utilized for *Pseudomonas* species to compete with other bacteria at colonization sites (94). Phenazine-producing strains of *P. aurofaciens* and *P. fluorescens* are better able to colonize the roots of wheat plants and persist in the rhizosphere than are phenazine-lacking mutants (78). In addition, it has been reported that phenazines secreted from *P. aeruginosa* PNA1, which is isolated from the rhizospheres of chic-kpeas, inhibited mycelial growths of phytopathogenic fungi, including *Fusarium oxysporum* f. sp. *ciceris* and *Pythium splendens* (3). On the other hand, pyocyanin-sensitive bacteria can coexist with pyocyanin-producing *P. aeruginosa* in a multispecies biofilm derived from soil extract (88). The presence of pyocyanin-resistant bacteria is necessary for the coexistence of these strains in a biofilm and this result showed that complicated interactions in a multispecies biofilm enabled microbial concomitance beyond the prey-predator relationship.

One of these phenazines has a key role in interactions between *P. aeruginosa* and a fungus *C. albicans*. When these two microorganisms were grown on an agar plate, a red pigment was synthesized. Gibson *et al.* (38) have shown that this pigment was derived from a precursor of pyocyanin, which is proposed to be 5-methyl-phenazinium-1-carboxylate (5MPCA), and accumulated exclusively within fungal cells. That red pigment was heterogeneous and hard to secrete into extracellular milieu, and definitely decreased fungal viability.

Phenazines also have a role as signal molecules in *P. aeruginosa*. While two AHL and PQS are synthesized in the late exponential phase, one phenazine, pyocyanin, is secreted in the stationary phase and upregulates genes that have previously been demonstrated to be controlled by quorum sensing (28). In addition, phenazines also control colony morphology in not only *P. aeruginosa* itself but also Gram-positive *Streptomyces coelicolor* (29). This effect is mediated through a transcriptional regulator, SoxR, in both bacteria. In this way, phenazine functions as an intercellular signaling molecule and enables crosstalk between different organisms.

The other important function of phenazines is as an electron shuttle which they promote microbial mineral reduction (42). This enables the bacterium to access to iron and various other nutrients, such as phosphate, trace metals, and organics that are associated with mineral phases. High-rate microbial electron transfer is utilized in microbial fuel cells (MFCs), which convert microbial metabolic energy obtained from their electron donor into electricity (21, 112, 140). Phenazines, particularly pyocyanin, secreted by *P. aeruginosa* are used as electron shuttles in other bacteria and support the production of electrochemical activity in MFCs (97, 98). In addition, analysis using MFCs revealed that phenazines also help the survival of *P. aeruginosa* PA14 under the conditions of oxygen limitation (139). In this way, electron transfer via phenazine is strongly associated with microbial behaviors in communities in both natural and engineered ecosystems.

## Rhamnolipids act as antimicrobials and cues

Rhamnolipids are a bacterial glycolipidic biosurfactants and prominent producers are the genus *Pseudomonas* and *Burkholderia*, but several bacteria belonging to *Actino-bacteria* and *Firmicutes* have also been reported to produce them (1). In *P. aeruginosa*, the synthesis of rhamnolipid is also under quorum-sensing regulation. Antimicrobial activity of rhamnolipids was observed against both Gram-negative species, including *Serratia*, *Enterobacter* and *Klebsiella*, and -positive species, including *Bacillus*, *Rhodococcus*, *Staphylococcus* and *Mycobacteium* (1). It has been considered that rhamnolipids intercalate into the biological membrane and cause destruction by permeabilizing effect (114), similar to that of synthesized surfactants. These antimicrobial activities of rhamnolipids have also been observed against a range of fungal species, but not against yeast (1, 39). Rhamnolipids are also involved in the detachment of biofilms. Boles *et al.* (7) showed that rhamnolipids promote the dispersion of *P. aeruginosa* cells from the biofilm, especially from the center of microcolonies. Rhamnolipids maintain open channels by affecting cell-cell interactions and the attachment of bacterial cells to the surfaces where bacteria seem to exploit intercellular interaction and communication to actively maintain these channels (23). One of the leading functions of rhamnolipids is the promotion of swarming motility (11). This may explain why rhamnolipids induce cell detachment from biofilms since swarming motility facilitates the detachment of cells in *P. aeruginosa* mature biofilm (10). Exogenous rhamnolipids interfere with the normal biofilm infrastructure in *P. aeruginosa* (23), and this detachment effect seems to be nonspecific, as the addition of rhamnolipid also disrupts *Salmonella typhimurium* and *Bordetella bronchiseptica* biofilms (50, 83). In this way, rhamnolipids have a role in not only antimicrobial effects but are also a cue to alter biofilm architecture.

## Exopolysaccharides are associated with interspecies interactions by forming biofilms or pellicles

It is known that *P. aeruginosa* secretes at least three distinct extracellular polysaccharides, alginate, Psl and Pel, and each of these exopolysaccharides has been found to be involved in biofilm formation (106). Psl polysaccharide is rich in mannose and galactose and its synthesis is encoded by *psl* cluster. Pel is a glucose-rich and cellulose-like polymer, and its synthesis is encoded by *pel* cluster. Recently Qin *et al.* (96) showed that the production of two expolysaccharides, Psl and Pel, inhibited staphylococcal growth and disrupted established *Staphylococcus epidermidis* biofilms. Indeed, cellulase-treated *P. aeruginosa* supernatant, and supernatant from *pelA*, *pslF* and *pelApslBCD* mutants, which are deficient in polysaccharide biosynthesis, diminished the disruption of *S. epidermidis* biofilms; however, it remains unknown whether these exopolysaccharides directly interact with *S. epidermidis* biofilm or affect other factors during the disruption of *S. epidermidis* biofilms. Since *P. aeruginosa* and *S. epidermidis* commonly coexist in clinical settings such as the surfaces of indwelling medical devices, these findings will contribute to the understanding of how *P. aeruginosa* inhibit the settlement of potential competitors.

In addition, surface-associated biofilms, Pel and Psl, are involved in pellicle formation in *P. aeruginosa* (143). Pellicles are known as microbial aggregations at the interface between air and liquid, and various aerobic microorganisms form pellicles to acquire oxygen effectively (142, 144). This kind of aggregation enables several bacterial species to coexist (40, 53–55, 144). According to these reports, an anaerobic cellulolytic bacterium *Clostridium straminisolvens* CSK1 could coexist in a multispecies culture including two pellicle-forming bacteria, obligate aerobe *Brevibacillus* sp. strain M1–5 and a facultative aerobe *Pseudoxanthomonas* sp. strain M1–3. Although the growth of M1–5 was inhibited by M1–3 when cultured together, the addition of CSK1 could alleviate the competitive interaction via a diffusible factor. In this way, the pellicle at the air-liquid interfaces may create an environment where multispecies bacteria can coexist through complex interactions.

## Fatty acid signals that affect biofilms formed by other species

Some fatty acids secreted by bacteria have a role as interspecies signaling molecules that modulate the microbial development of different species. It is known that a plant pathogen *Xanthomonas campestris* produces a diffusible signal factor (DSF: cis-11-methyl-2-dodecenoic acid) (136). DSF not only controls biofilm formation and virulence in *X. campestris* but also alters the morphology of other bacteria. Indeed, Ryan *et al.* (105) showed that DSF secreted from *Stenotrophomonas maltophila* causes an alteration of *P. aeruginosa* biofilm architecture and increases the tolerance against antibiotic polymyxin by a sensor kinase PA1396. *S. maltophila* exists ubiquitously in various environments, as does *P. aeruginosa*, and it can be considered that this interaction is relevant in diverse niches including the rhizosphere of plants and the cystic fibrosis lung. Recently Twomey *et al.* (132) indicated that several kinds of cis-2-unsaturated fatty acids are detected in sputum from CF patients, where clinical *P. aeruginosa* isolates retained the ability to sense and respond to those fatty acids, and the virulence and antibiotic resistance of *P. aeruginosa* are enhanced by them. Since the presence of these signal molecules correlates with polymicrobial infection involving *Burkholderia* and/or *Stenotrophomonas* species together with *P. aeruginosa*, this evidence supports the importance of fatty acid signals in interspecies interactions at actual chronic infection sites.

*P. aeruginosa* also secretes a fatty acid signal, cis-2-decenoic acid (Fig. 1F), that is structurally similar to DSF. Interestingly, cis-2-decenoic acid is capable of dispersing biofilms formed by not only *P. aeruginosa* but also *E. coli*, *Klebsiella pneumoniae*, *Proteus mirabilis*, *Streptococcus pyogenes*, *Bacillus subtilis*, *S. aureus*, and the yeast *C. albicans* (24). Collectively, this class of short-chain fatty acid signaling molecules acts as a cell-to-cell communication molecule that particularly alters biofilm structures in various bacteria, including *P. aeruginosa*.

## Type VI secretion system virulence effectors lyse other bacteria

Several pathogens including *P. aeruginosa* use T6SS to transport proteins into the environment or host cells in response to external stimuli (67). T6SS genes in *P. aeruginosa* are found in three pathogenic islands, hemolysin co-regulated protein secretion island-I (HSI-I), HSI-II, and HSI-III (95). These gene expressions are under the control of QS; Las and Rhl positively regulate HSI-2 and HSI-3 gene clusters while Las also negatively regulates the HSI-1 gene cluster (66). Recently, the Mougous group has discovered that T6SS is an ingenious weapon for *P. aeruginosa* to inject specific proteins into other Gram-negative bacteria such as *E. coli*, *P. putida* and *Burkholderia thaillandensis* (47, 104). At least three proteins are exported through T6SS in *P. aeruginosa*: Tse1, Tse2 and Tse3 (47, 87). Tse2 protein is the toxin component and arrests the growth of Gram-negative bacteria, as well as eukaryotic cells such as *Saccharomyces cerevisiae* (47). Tse1 and Tse3 are enzymes that degrade bacterial peptidoglycan, but *P. aeruginosa* has a protective mechanism against lysis to localize immunity proteins Tsi1 and Tsi3 (104). It is considered that the function of Tse1 and Tse3 is not just to advance the needle, but also to kill the target cells. As explained, the T6SS system is likely an effective apparatus that enables the delivery of killer proteins to competitive bacteria directly.

## Membrane vesicles

As noted above, *P. aeruginosa* produces MVs and has been studied as a model bacterium for understanding the biophysical characterization and biogenesis of bacterial MVs (6, 75, 119–122, 124). MVs are typically released from Gram-negative bacteria, which range from 50 to 250 nm in diameter and are mainly composed of phospholipids, lipopolysaccharide (LPS), outer membrane proteins and periplasmic components. MVs are one of the mechanisms for secretion that is pivotal for delivering certain toxic proteins. *P. aeruginosa* MVs contain secreted virulence factors for killing host cells or other bacteria, including protease, elastase, hemolysin, phospholipase C, alkaline phosphatase, Cif, as well as other antibacterial factors, such as murein hydrolases. In addition, *P. aeruginosa* MVs possess PQS and extracellular DNA on their surfaces. In this way, MVs package several concentrated substances and have a role to convey them to others.

Because *P. aeruginosa* MVs contain antibacterial quinolones (73) and murein hydrolase (69), they possess strong microbial activity against both Gram-negative and Gram-positive bacteria. As noted above, PQS can inhibit the growth of other bacteria and the majority of these effects are thought to be due to its iron-chelating activities (127). Murein hydrolase can cleave the covalent bonds in peptidoglycan. Indeed, Li *et al.* (68) showed that MVs derived from 15 other Gram-negative bacteria have the ability to lyse a variety of Gram-negative and Gram-positive bacteria; in particular, *P. aeruginosa* MVs can lyse the broadest spectrum of other bacteria among those that have been tested. In addition, encapsulation of gentamicin into MVs seems to be a strategy for enhancing competition in microbial communities. The MVs secreted following exposure to gentamicin possess a high concentration of this antibiotic and have enhanced lytic effects against other bacteria (2, 71). These characteristics of having strong microbial activities contribute to defeat competitors in polymicrobial environments.

MVs are also tools for the secretion of extracellular DNA. Renelli *et al.* (101) showed that extracellular DNA is not only associated with the surface of *P. aeruginosa* MVs, but is also localized inside MVs. Since DNA-contained MVs are not sensitive to DNase, DNA contained in MVs may be stored for a long time in the environment (101). The transfer of DNA from one strain to another via MVs has been reported in *E. coli* O157:H7 (62), but that phenomenon has not been found between *P. aeruginosa* species. One reason is thought to be that *P. aeruginosa* is not known to be naturally transformable. In contrast, Chiura’s group has reported that virus-like particles (VLPs), which are another type of small particles secreted from various bacteria in ocean and thermal environments, have a role in broad-host range gene transfer with extremely high transduction frequency (14–18, 79). While MVs are encapsulated from the bacterial outer membrane (containing proteins and phospholipids), the outer envelope of VLPs is composed of proteins. It is still possible that *P. aeruginosa* MVs contribute to DNA transfer to naturally transformable bacteria in the environment and, if so, MVs would play an important role in horizontal gene transfer for bacterial evolution as well as VLPs in environmental microbial consortia.

## Micro-fabrication technologies as a tool for studying interspecies communication

In the last decade, application of micro-fabrication technology has started to create unique opportunities in the study of cell-cell communication. micro-fabrication technologies that were originally developed for semiconductor chip production are now being applied to create a micro-scale (mm-nm scale) culture/analysis tool for microbiology, which is also referred to as micro-fluidic device or Micro-Total-Analysis-Systems (μTAS). Those micro-fabrication technologies provide many advantages over existing macro-scale experimental setups, enabling us to address questions that are difficult to handle with conventional methods. Micro-fabrication technology has also been applied to the studies of *P. aeruginosa*, addressing chemotaxis (52), biofilm formation (59, 147, 148), growth measurement (146) and screening of a multidrug efflux pump inhibiter (49, 76). This section will review cell-cell communication studies that employed various types of micro-fabrication technology (from simple droplet creation to complicated micro-machinery) and further discusses the potential application of such micro-fabrication technology to studies of interspecies communication between *P. aeruginosa* and other species.

**Fig. 5:**
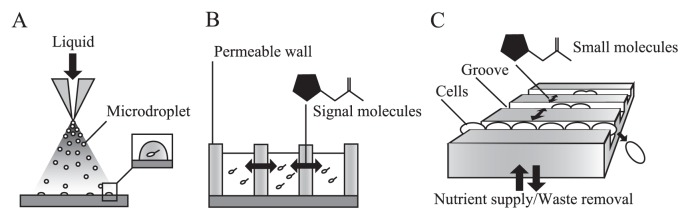
(A) Concept of the production of a micro-droplet containing a single cell. Liquid mixture containing diluted cells was sprayed as an aerosol onto a glass substrate. (B) Permeable wall made with poly(ethylene glycol) diacrylate (PEGDA) hydro-gel facilitates the free exchange of signaling molecules between neighboring wells while spatially segregating two cultures. (C) Concept of “single cell chemostat”. Bacterial cells are grown in a sub-micron groove patterned on agarose pads. The thin groove forces a bacterial cell to form a linear micro-colony along with the groove as the starting single cell for duplication. Nutrients are supplied from agarose pads, and metabolites are also removed through agarose pads, forming the stable condition.

A study by Carnes *et al.* (13) that demonstrated “single-cell quorum sensing” shows an example of the unique opportunity provided by the simplest micro-fabrication technology. Although the term “single-cell quorum sensing” sounds like a contradiction, in this study, the authors demonstrated the effect of confinement on quorum sensing. The authors encapsulated a single *S. aureus* cell in a micro-droplet of lipid-silica with a diameter of less than 20 mm. These droplets containing a cell were obtained by spraying the mixture liquid of lipid silica and cells as aerosol (Fig. 5A). Within the tiny confined micro-droplet, *S. aureus* was shown to be capable of initiating quorum sensing at the single-cell level due to the accumulation of signaling molecules in a confined space. The authors argue the relevance of such confinement to the situation within an endosome or phagosome during infection. This study well demonstrated that the threshold cell number (quorum) at which quorum sensing occurs is highly environment dependent, reminding us that the same might hold true for interspecies communications.

In the above example, micro-fabrication technologies were used to limit diffusion by creating micro-droplets. Contrary, Flickinger *et al.* (35) utilized micro-fabrication technologies to allow spatially isolated biofilms to interact via cell-cell communication. The authors fabricated an array of small-culture chambers embossed in a layer of permeable poly(ethylene glycol) diacrylate (PEGDA) hydro-gel, which facilitates the free exchange of signaling molecules between neighboring culture chambers via a permeable wall (Fig. 5B). Such micro-culture chambers as used in this study are made by casting the materials in a mold; therefore, by changing the material for the casting, the diffusivity inside the micro-channel can be easily controlled. Using this device, the authors showed that the signaling molecule from a neighboring culture chamber enhances the growth of *P. aeruginosa* biofilms. As demonstrated in this study, such a micro-culture chamber with a permeable wall allows the culture of multiple groups of organisms in a “spatially separately, yet chemically connected” manner, which is ideal to test the interaction between different species via cell-cell communication. Such control of diffusivity will be one of the most useful features of micro-fabrication technologies in the study of interspecies cell-cell communication of *P. aeruginosa* via AHLs, PQS and other chemical signals (118, 127, 145).

Although hydro-gel is frequently used when fabricating simple micro-culture devices that require permeable walls, silicone-based material (poly-dimethyl-cyloxane: PDMS) is preferred when fabricating a more complicated or finer structure, because of the more rigid and stable nature of PDMS. Park *et al.* (91) fabricated a micro-scale maze within the PDMS layer. The authors observed that *Vibrio harveyi* cells, which were initially evenly distributed within the PDMS maze, eventually spontaneously gathered in a dead end in the maze at high cell density, and readily started emitting fluorescence – initiated quorum sensing. The authors showed that bacterial gathering at the dead end was driven by chemotaxis to the amino acid that was secreted by *V. harveyi* cells themself, showing that chemotaxis provides an important mechanism for establishing the high local cell densities required for quorum-dependent interactions. This study demonstrates the usefulness of micro-fabrication technologies to investigate bacterial cell-cell communication behavior in complicated topology that has been impossible with a conventional culture method.

The PDMS molding technique also enables the construction of a further complicated “on-chip” micro-liquid handling device that focuses on controlling tiny quantities of liquids. Hong *et al.* (46) fabricated a micro-liquid handling device that has a culture-chamber to grow biofilm and multiple inlets to deliver stimulants to the culture chamber. With the micro-liquid handling device, the authors could freely add AHL or AHL-producing cells as stimulants to the biofilm of genetically engineered *E. coli* growing in the culture chamber and observed the biofilm’s response by confocal microscopy in real time. The genetically engineered *E. coli* genome was supplied with a synthetic quorum-sensing circuit that is programmed to undergo biofilm dispersal in response to 3-oxo-C12-HSL. The micro-liquid handling device served as an ideal platform for the authors to test if the genetically engineered *E. coli* actually undergoes biofilm dispersal as programmed in response to the addition of HSL or HSL-producing cells. Such a micro-liquid handling device would also be an ideal platform to investigate the effect of endogenous cell-cell signaling molecules in interspecies communication between *P. aeruginosa* and other species.

As reviewed in this section, micro-fabrication technologies have already provided many unique experimental set-ups for cell-cell communication, such as confinement, a permeable wall, complicated topology and real-time observation of the response to stimulation. On the other hand, there are micro-fabrication technologies yet to be applied to the study of cell-cell communication, as described below.

This example is a micro-culture device referred to as a “single-cell chemostat” or “mother machine” (84, 137). The basic idea here is to grow a single bacterial cell in the sub-micron groove patterned on agarose pads (Fig. 6C). The thin groove forces a bacterial cell to form a linear micro-colony along the groove as the starting single cell for duplication. Nutrients are supplied from agarose pads, and metabolites are also removed through agarose pads, forming the stable condition. Therefore, this culture device is called a “single-cell chemostat” and enables the growth kinetics of a single cell to be tracked for over 30–40 generations. An interesting point here is that a single groove is not patterned on the agarose pad, but a parallel array of grooves; therefore, it can be observed very precisely how cells in neighboring grooves affect each other’s growth. The authors confirmed this idea by growing a community of *E. coli* auxotophs that can complement each other’s amino acid deficiency. It would be very straight forward to expect such a culture device to be applied to observe how *P. aeruginosa* and other species affect each other’s growth via cell-cell communication, as PQS, AHLs or its degradation products are known to affect respiration and growth in *P. aeruginosa*.

Although micro-fabrication technologies have been applied to the study of quorum-sensing and cell-cell communications, examples are rare that apply micro-fabrication technologies to the study of interspecies communication between *P. aeruginosa* and other species. As seen in studies using micro-droplets and the micro-maze, even the very simple application of micro-fabrication technologies can suggest surprising novel ideas. Thus, we can expect that the use of micro-fabrication technology will also bring new aspects to the study of interspecies communication between *P. aeruginosa* and other species.

## Concluding remarks

Although global analysis has developed knowledge of microbe communities in natural environments, this analysis has not reached the understanding of microbial interactions. Over past years, a stronger emphasis has been placed on discovering these interactions between lower life forms by co-culturing several species of microbes. This review revisits the microbial relationship associated with *P. aeruginosa*, which has sophisticated functions to interact with other bacteria, including both antimicrobial effects and cross-talk. In either case, most functions are under quorum-sensing regulation in *P. aeruginosa*. In contrast, other bacteria have multiple strategies to inhibit *P. aeruginosa* quorum sensing to cope with its antimicrobial effects; however, we may yet only have had a glimpse into how microbes interact with each other in natural environments. Microbial interspecies interactions must be much more complex and sophisticated than hitherto realized. New technical tools such as micro-devices may enable us to further tackle this question. We hope this review will contribute to the study of the evolution of the cooperation and social behavior of microbes.
